# Integrating music information retrieval into performers’ reflective practice: an interdisciplinary approach to performance learning

**DOI:** 10.3389/fpsyg.2026.1780363

**Published:** 2026-03-19

**Authors:** You Jin Kim, Moo Kyoung Song

**Affiliations:** 1 Graduate School of Education, Music Education, Kyung Hee University, Seoul, Republic of Korea; 2 College of Music, Music Theory, Yonsei University, Seoul, Republic of Korea

**Keywords:** music information retrieval, music performance analysis, performer’s expressivity, *sanjo*, vibrato analysis, virtuoso’s performance

## Abstract

Music performance analysis increasingly aims to address measurable acoustic features alongside performers’ ways of understanding, internalizing, and applying expressive knowledge within culturally grounded traditions. Although Music Information Retrieval (MIR) techniques have advanced considerably in their capacity to quantify expressive parameters such as vibrato extent and rate, much of this scholarship has continued to privilege technically oriented examinations of performance This study examines expressive vibrato as a locus where performer individuality, lineage-based style, and reflective learning converge. Focusing on a historically informed *sanjo* recording by the virtuoso Kim Juk-pa, the study integrates music information retrieval (MIR)–based acoustic analysis with professional performers’ interpretive reflections. Quantitative analyses revealed systematic variation in vibrato rate and extent across melodic structures and *jo* idioms (i.e., mood or affective states), indicating that vibrato shaping is closely aligned with expressive and structural contexts, while no clear interactive effects emerged between the two parameters. Analysis of coefficients of variation further revealed stable internal relationships among vibrato parameters that characterize the expressive logic of the virtuoso’s *sanjo*. Performers interpreted these findings as reflective resources rather than prescriptive technical norms; the vibrato profiles rendered tacit, embodied lineage knowledge more explicit, enabling performers to re-engage with stylistic archetypes, refine micro-expressive control, and negotiate the balance between fidelity to inherited style, pedagogical influence, and personal artistic identity. The analytical profiles were described as landmarks within a broader expressive terrain in support of stylistic recentering while preserving creative agency. The study demonstrates how MIR-based music performance analysis can acquire pedagogical and artistic significance when embedded within performers’ culturally situated learning practices and offers a transferable framework for reflective performance learning across musical genres.

## 1 Introduction

Mastery of musical performance does not emerge solely from technical execution but also hinges on other things such as the performer’s capacity to shape expressive content within culturally informed musical structures (Sloboda, 2000). Performance as an artistic and cognitive endeavor is contingent upon a complex interplay of genre-specific musical knowledge and culturally embedded stylistic conventions. As Hunter and Broad (2017) assert, a nuanced understanding of genre characteristics is indispensable for analyzing performers’ interpretive strategies and expressive decisions.

The shaping of expressivity unfolds through performers’ internalized engagement with stylistic idioms and culturally sedimented aesthetic codes. Expressive agency emerges within historically constituted frameworks that both enable and constrain interpretive possibility, situating performers within lineages of meaning that precede and exceed individual intention. In this sense, expressivity is constituted through a dynamic negotiation between personal artistic orientation and collectively inherited expressive archetypes embedded in genre traditions (Kim and Song, 2023). Such negotiation is neither purely subjective nor mechanically determined; it is structured by the symbolic systems through which musical practices acquire coherence, authority, and cultural legitimacy. The ontological framing of music performance, including the symbolic and ideologically encoded meanings of musical idioms, serves as a foundational lens through which performer individuality can be understood. This conceptual grounding becomes particularly salient when examining contemporary developments in technology-mediated performance analysis and training, which operate within the aesthetic and ontological frameworks that shape expressive meaning.

In a seminal contribution to technology-mediated performance training, Timmers and Sadakata (2014) argue that visual feedback grounded in performance measurement techniques can deepen performers’ intrinsic sensory awareness by externalizing microstructural variations in timing, dynamics, and articulation. They characterize such visualizations as analytically selective abstractions that foreground particular parametric dimensions while delimiting the full complexity of expressive action. By rendering temporal shaping perceptually salient, these systems bring tacit performative configurations into reflective awareness and encourage more deliberate engagement with expressive processes. Empirical work within the Practice Space framework further demonstrates that augmented feedback can refine timing nuance and stylistic control, especially when structured to support exploratory engagement. In parallel, the authors outline design principles that condition pedagogical efficacy, including the balance between analytic specificity and holistic evaluation and the careful regulation of feedback frequency. They also draw attention to the risk that continuous real-time feedback may foster dependency and weaken reliance on intrinsic sensory cues, while narrowly defined parameter visualizations risk privileging mechanical regularity over interpretive latitude. Such observations underscore that visual feedback systems carry implicit aesthetic orientations and that their educational value depends on contextual alignment with artistic aims.

Complementary research on computer-assisted feedback systems further indicates that performers’ engagement with technological tools is shaped by interpretability, specificity, and explanatory framing (Karlsson et al., 2009). Algorithmic systems can quantify acoustic features associated with expressive communication; however, performers assess feedback primarily in relation to its relevance for musical decision-making rather than its technical precision. Visualization studies likewise show that expressive parameters including tempo and loudness can be represented within integrated spatial models that illuminate large-scale shaping processes and inter-parameter relationships (Langner and Goebl, 2003). Performance measurement acquires pedagogical significance when acoustic data are structured in ways that resonate with performers’ embodied knowledge and stylistic understanding.

From a broader theoretical perspective, computational models of expressive music performance conceptualize timing and dynamics as systematic and analyzable phenomena embedded within musical architecture (Widmer and Goebl, 2004). Contemporary surveys of educational technologies reveal that many applications remain focused on pitch and rhythmic accuracy, with comparatively limited engagement with timbral sensitivity, articulatory refinement, and culturally situated expressive gestures (Acquilino and Scavone, 2022). These considerations echo earlier analyses of technology in performance learning and reinforce the argument that digital tools attain pedagogical legitimacy only when they operate within the social ecology and interpretive traditions that structure musical practice (Walls, 1997).

These perspectives underscore the importance of positioning performance measurement technologies as reflective instruments within an interpretive ecology. Within this trajectory of technology-enhanced performance learning, Music Information Retrieval (MIR) represents a further methodological refinement. MIR enables systematic modeling of expressive parameters with a level of acoustic resolution that makes subtle microstructural variation available for close examination. MIR-based vibrato analysis, in particular, may be understood as a structured analytical interface through which lineage-specific expressive gestures become acoustically traceable and open to sustained interpretive inquiry. When incorporated into performer-centered practice and dialogic reflection, MIR functions as a resource for deepened stylistic awareness.

Within music performance research, certain expressive parameters have proven especially suitable for systematic investigation. Vibrato occupies a prominent position among them as a finely calibrated expressive resource whose fluctuations in pitch contour and oscillatory behavior directly shape the perceived character of a performance (Lerch et al., 2020). Because vibrato operates at the intersection of technical control and interpretive intention, it provides an analytically tractable entry point into the study of musical expressivity. Its measurable acoustic properties make it particularly responsive to empirical inquiry, while its perceptual salience ensures interpretive significance.

A substantial body of scholarship has examined vibrato as a communicative medium through detailed acoustic analysis, demonstrating how variations in oscillation patterns contribute to perceptions of individuality and expressive nuance (Juslin and Timmers, 2010). Related investigations further show that vibrato functions as a marker of stylistic distinction across performers and traditions, shaping the aesthetic profile of a rendition in ways that listeners readily perceive (Delviniotis et al., 2013; Prame, 1994, 1997; Widmer, 2005; Zhang et al., 2017). Through these studies, vibrato emerges as a locus where technical execution becomes inseparable from interpretive stance.

The annotation of vibrato parameters has gained methodological relevance in performance studies. Widmer et al. (2003) suggest that vibrato can disclose aspects of a performer’s “special art,” pointing to its capacity to reflect both cultivated technique and interpretive orientation. In this sense, vibrato functions as an expressive indicator that links acoustic microstructure to artistic identity. Its shaping reflects historically situated stylistic conventions alongside the performer’s own aesthetic disposition. As Ornoy and Cohen (2018) observe, vibrato often serves as an audible signature through which stylistic identity becomes perceptible. Systematic examination of vibrato within culturally grounded performance practices therefore offers a focused pathway for understanding how expressive traditions are internalized, negotiated, and rearticulated in virtuosic interpretation.

Despite the development of Music Information Retrieval (MIR) techniques for analyzing expressive parameters such as vibrato extent and rate, much of this work has remained largely focused on technically oriented observations of performance parameters (Lerch et al., 2020). While such analyses have contributed substantially to the objective modeling and classification of musical expressivity, their pedagogical and experiential relevance for performers has received comparatively limited attention. That is, engagement with culturally embedded and aurally transmitted performance traditions remains comparatively limited, particularly in contexts where stylistic knowledge circulates through apprenticeship. Therefore, further clarification is needed regarding how quantitatively derived MIR representations can be meaningfully situated within performers’ reflective and performance-learning practices processes beyond its technical accuracy. For this reason, the present study addresses a longstanding divide between quantitative performance analysis and humanistic traditions of performance learning, which foreground embodied knowledge, lineage transmission, and interpretive agency.

MIR’s capacity for high-resolution acoustic analysis presents an underutilized opportunity. When situated within performer-centered inquiry, precise measurement can serve as an analytical resource that supports performers’ reflective understanding of their own expressive strategies. By making microstructural variation perceptually and conceptually explicit, MIR has the potential to inform interpretive decision-making and deepen performers’ articulation of artistic individuality across diverse musical cultures.

The present research advances this perspective by framing MIR-based vibrato analysis as a mediating instrument within reflective practice. Through the integration of acoustic profiling of a *sanjo* virtuoso’s vibrato with in-depth interviews with professional performers, the study demonstrates how computational outputs can catalyze heightened awareness of expressive nuance, stylistic inheritance, and interpretive positioning within a lineage-based tradition. In doing so, MIR is repositioned from a tool of external analysis to a dialogic interface that facilitates critical engagement with the expressive foundations of performance.

By engaging with detailed vibrato-specific annotations derived from a master’s performance of *sanjo*—a genre defined by lineage and virtuosity—performers are afforded an opportunity to embrace empirical findings appraised through the lens of lived artistic practice. This process situates music performance analysis within a dialogic space in which data-driven insights are transformed into pedagogically meaningful knowledge. Such expansion becomes meaningful as a descriptive and instructional framework that informs and shapes the evolving practice of expressive performance within culturally rooted traditions. Additionally, Western classical traditions typically foreground the interpretive autonomy of the performer in realizing composed works (Solomonova et al., 2023), whereas performance traditions rooted in virtuoso-driven lineages emphasize the emulation, internalization, and refinement of stylistic models transmitted across generations (Kim and Song, 2023). In these contexts, the performer’s expressive output is a reflection of creative spontaneity aligned with a dialogic reconstruction of culturally revered prototypes. This study also adopts the perspective of the aforementioned performance traditions to investigate how genre-specific expressive gestures—exemplified by vibrato—are acoustically encoded and interpreted, and how professional performers integrate such analytical insights into their own processes of artistic mastery.

In summary, this study situates the acoustic analysis of vibrato in *sanjo* as a reflective resource through which contemporary performers may re-examine and refine their understanding of expressive lineage. *Sanjo*, a traditional Korean solo instrumental genre, is characterized by highly ornamented melodic elaboration within cyclical rhythmic frameworks and is deeply rooted in individualized stylistic transmission across virtuoso lineages. Among its representative forms, *gayageum sanjo*—performed on the Korean zither—foregrounds nuanced left-hand techniques that shape microtonal inflection, ornamental contour, and vibratory modulation. The interaction between right-hand plucking and left-hand pitch manipulation generates a distinctive vibrato whose depth and oscillatory profile vary systematically across modal contexts, *jo* such as *u-jo*, *p-jo*, and *g-jo*. These modal inflections contribute to differentiated expressive atmospheres and aesthetic identities within performance practice (Kim, 2016). By engaging this vibratory microstructure through acoustic analysis, the present study approaches a virtuoso’s *sanjo* as an interpretive field in which computational insight and performer reflection converge.

## 2 Multidimensional foundations of musical performance expressivity

Musical expressivity in performance resists a simple definition, requiring contextualization within both cognitive and cultural dimensions. Its characterization is inherently multifaceted and involves nuanced interactions between the structural, emotional, and embodied aspects of musical expression. To address this complexity, Juslin et al. (2001) advanced a foundational framework for research on expressive music performance, emphasizing the systematic identification of sources of performance variability, the integration of multiple expressive mechanisms, and the development of computational approaches to modeling expressive behavior. Within this framework, four interacting components—generative, emotional, random, and motion-based mechanisms—serve as explanatory constructs for expressive variation. The generative component encompasses expression driven by the structural and stylistic features of the music, while the emotional component accounts for the deliberate modulation of acoustic cues to convey affective intent. Random variation introduces micro-level unpredictability into performance, simulating spontaneity—which is accounted for in the random component of the framework. The movement component of the framework captures the natural dynamics of the bodily gestures involved in sound production. In the emotional domain, in particular, there has been significant scholarly emphasis on the ways in which performers shape acoustic parameters such as timing, dynamics, and timbre to communicate affect to listeners (Gabrielsson and Juslin, 1996; Gabrielsson and Lindström, 2010). This communicative process is bidirectional, as performers encode expressive cues based on intended emotions, which listeners then decode through perceptual and cultural schemas (Juslin and Lindstrom, 2016; Juslin, 2019). The success of this communication is dependent on both the clarity of the expressive intent and the interpretive competencies of the listeners.

Notably, musical expressivity in performance is shaped by culturally specific frameworks. As emphasized by Blacking (1973), the emotional meanings attributed to music are not inherent but are learned and enacted within particular cultural contexts. Extending this perspective, Turino (2008) argues that musical expressivity is fundamentally grounded in cultural participation and identity, with performers drawing upon culturally recognized expressive signs to render affective meaning socially intelligible. Through this interplay, performers’ expressive agency is both enabled and constrained by culturally encoded idioms, which mediate how emotionally nuanced and stylistically refined performances are realized.

## 3 Vibrato in performers’ expressivity

The study of vibrato through sound-profile analysis has substantially enriched the methodological landscape of music performance research by enabling precise quantification of its acoustic characteristics. Yet, to investigate vibrato as an expressive phenomenon, a coherent and operational conceptualization is essential. One of the earliest and most widely cited definitions is offered by Seashore (1937), who described vibrato as a “periodic pulsation of pitch, loudness, timbre, or in combination” (p. 30). Building on this foundational view, subsequent research has approached vibrato as a multidimensional acoustic event. Early empirical work by Sundberg (1987) demonstrated that vibrato consists primarily of quasi-periodic oscillations in fundamental frequency, often accompanied by coordinated variations in intensity. Complementing this, Prame (1994, 1997) provided detailed measurements of vibrato rate and extent across singers and stylistic contexts, revealing systematic relationships between pitch modulation patterns and expressive intent.

The field of music performance study has moved beyond manual annotation toward automated detection systems capable of identifying psychoacoustic features, such as vibrato rate and vibrato extent. These features often align closely with the expressive strategies employed by individual performers, which are shaped not only by personal style but also by culturally embedded performance norms and structural cues within the music (Delviniotis et al., 2013; Prame, 1994, 1997; Timmers and Desain, 2000). With the development of algorithmic detection tools tailored for monophonic textures (Bogdanov et al., 2013), vibrato analysis has become increasingly scalable and objective, supporting broad comparative studies and more consistent analytical outcomes (Goebl et al., 2008). Complementing these detection systems are filtering techniques such as harmonic–percussive source separation (Fitzgerald, 2010), which allow researchers to isolate relevant sonic materials while reducing interference from surrounding textures. These advances refine the analytic process and enhance the reliability of vibrato measurements, particularly in complex or polyphonic recordings. Specifically, methodological and technological developments delineate a highly refined and multidimensional understanding of vibrato as a form of expressivity shaped by the performer’s interpretive intent and the structural affordances embedded within the music itself—a key direction taken up in the present study.

## 4 The present study

The present study seeks to connect a sound-profile analysis of vibratos in a *sanjo* virtuoso’s performance with contemporary *sanjo* performers’ reflections on the outcomes of that analysis. The study examines the acoustic parameters of vibrato segments in a historically renowned recording to illuminate the stylistic vibrato techniques employed by a *sanjo* virtuoso. The *jo* idiom—defined in traditional Korean music as a culturally grounded melodic and affective framework, including *u-jo*, *p-jo*, and *g-jo*—structures pitch organization, modal contour, and expressive character within performance (Kim and Song, 2023). The examination of music-specific expressive features, including vibrato within a particular genre, has gained scholarly prominence due to ongoing discussions concerning the culturally situated and musically contingent nature of expressiveness in performance.

Methodologically, the study proceeds through phases centered on a detailed analysis of recorded material. The process begins with the identification of vibrato segments, followed by the selection of analytical tools appropriate for investigating their acoustic properties. In parallel, the study incorporates an in-depth reflection of current *sanjo* performers aimed at refining expressive strategies in their own practice based on the analytical outcomes of Phase 1.

This study is guided by two research questions:

(1) What defining characteristics of vibrato emerge in relation to different melodic lines and the *jo* idiom?
(2) How do current *sanjo* performers interpret and incorporate the analytical findings from Phase 1 to their learning and performance processes?

## 5 Methods

This study comprises two interconnected phases: a quantitative analysis of the acoustic characteristics of selected vibrato samples conducted in Phase 1, and a qualitative investigation into how these features are interpreted and applied by current *sanjo* performers conducted in Phase 2. In the first phase, vibrato samples were extracted from an archival *sanjo* recording, and the acoustic parameters of these vibrato samples were analyzed using signal processing techniques. This signal analysis was complemented by in-depth interviews with current professional performers who specialize in the style of Kim Juk-pa’s *sanjo*, allowing for an integrated understanding of vibrato as both a measurable parameter and an expressive resource situated within a specific musical tradition.

### 5.1 Phase 1: acoustic characterization of vibrato

#### 5.1.1 Vibrato extraction and estimation

A central focus of this study is the accurate estimation of pitch fluctuation and vibrato rate in the *sanjo* performances of Kim Juk-pa. Because her *sanjo* was performed on the *gayageum*—a traditional 12-string zither-like instrument—particular attention was given to the unique acoustic properties of its string sound, including its rich overtone structure and subtle pitch inflections. For our analysis, we selected a historically significant archival recording of Kim Juk-pa that is widely regarded as a posthumous masterpiece as the primary data source. This recording provides an exemplary case for examining vibrato within the stylistic context of traditional Korean string performance.

Employing music information retrieval technology, the archival audio recording was processed using Essentia, an open-source audio analysis library that offers a wide array of algorithms for the automated extraction of musical features—including vibrato segments (Bogdanov et al., 2013). Notwithstanding its robust capabilities, the Essentia vibrato detection algorithm failed to reliably identify the onset and offset boundaries of vibrato in the selected Kim Juk-pa *sanjo* performance. This limitation appears to stem from the culturally specific intervallic structures and expressive timing characteristics of *sanjo* vibratos, which diverge from the more regularized vibrato profiles assumed in the design of the algorithm. To overcome this constraint, the lead researcher—being a *sanjo* performer—conducted manual annotations of the vibrato segments using Sonic Visualiser (Cannam et al., 2010). This process allowed for detailed temporal labeling, which was essential for the accurate extraction of the acoustic parameters. Each vibrato instance was segmented and treated as an independent unit of analysis, yielding a total of 119 vibrato samples drawn from 100 melodic units. These melodic units encompassed a range of pitch contours—including ascending, descending, and static phrases—thus reflecting the expressive breadth inherent in the *sanjo* tradition. To accurately isolate the vibrato signal from the recording, we experimented with Demucs, an AI-based source separation model; however, this approach is primarily optimized for popular-music instrument configurations (e.g., drums, bass, vocals) and proved ineffective for separating sources in Korean traditional music contexts. Accordingly, we employed the harmonic–percussive source separation (HPSS) function implemented in Librosa (Fitzgerald, 2010; McFee et al., 2015), a Python-based library for music and audio analysis. While not entirely artifact-free, this approach effectively attenuated the percussive components of the accompanying barrel drum, thereby enabling the reliable extraction of continuous pitch contours essential to the analysis of the tonal content in *sanjo.*

Monophonic pitch estimation was performed using the pYIN algorithm (Mauch and Dixon, 2014), a probabilistic extension of the YIN pitch detection method that has become a well-established tool for tracking pitch trajectories in monophonic audio signals. Vibrato peaks and troughs were then identified via a peak-and-valley detection process applied to the pitch contour. To ensure data reliability, segments exhibiting irregular vibrato patterns were visually inspected and filtered. Vibrato curves with excessive waveform distortion were excluded from the dataset based on the degree of deviation from the expected sinusoidal patterns.

The number of crests (i.e., the highest pitch points within each vibrato cycle) and troughs (i.e., the corresponding lowest pitch points) in the final dataset were used to calculate the instantaneous vibrato rate and vibrato extent. These parameters served as the basis for subsequent acoustic analyses, providing a quantifiable lens through which to examine the expressive and stylistic features of Kim Juk-pa’s *sanjo* performance.

We employed a two-way factorial analysis of variance (ANOVA) to independently assess the effects of *jo* type and melodic line patterns on two key vibrato parameters: vibrato extent and vibrato rate. The *jo* types were classified as *u-jo* (majestic), *p-jo* (peaceful or joyful), and *g-jo* (melancholic or sad), as explicitly indicated in the transcribed score of Kim Juk-pa’s *sanjo* performance (Kim and Song, 2023). The melodic line patterns were similarly classified into three categories: ascending, descending, and static. To satisfy the assumptions of ANOVA—particularly the homogeneity of variance assessed using Levene’s test—we employed several data transformation techniques. To satisfy the assumptions of ANOVA—particularly the homogeneity of variance assessed using Levene’s test—we conducted a series of data screening and transformation procedures following the guidelines outlined by Field (2005). First, the distributions of vibrato extent and vibrato rate were inspected visually using histograms and Q–Q plots, and statistically evaluated using tests of normality. Second, homogeneity of variance across conditions was assessed using Levene’s test, which indicated violations of this assumption in the raw data. Third, several transformation techniques recommended for positively skewed data (including logarithmic, square-root, and reciprocal transformations) were systematically applied and compared. Each transformed dataset was re-evaluated in terms of distributional normality and variance homogeneity. Among these approaches, applying a square-root transformation to vibrato extent ( $\sqrt{\mathrm{ve}}$ ) and a power transformation with exponent 1.5 to vibrato rate ( $\sqrt[{3}]{\mathrm{vr}})$ yielded the greatest improvement, resulting in reduced skewness and acceptable homogeneity of variance. Accordingly, all subsequent ANOVA analyses were conducted on these transformed data to enhance the reliability and validity of the statistical results.

#### 5.1.2 Quantitative data analysis results

**Figure 1:**
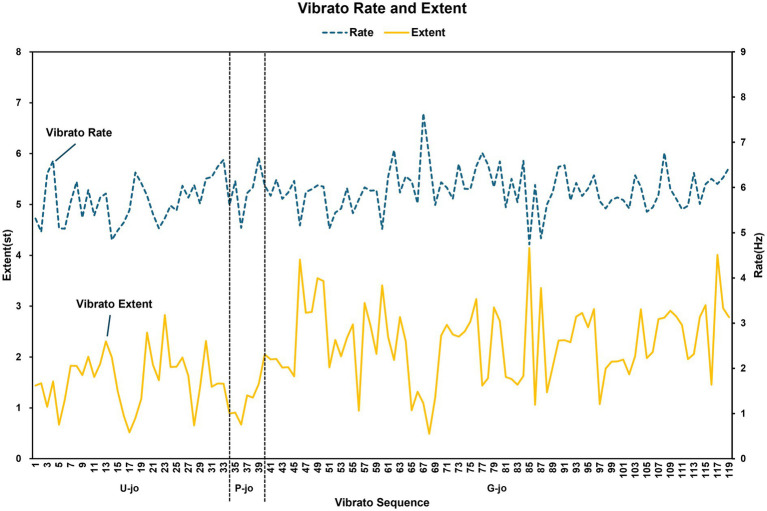
Visualization of the distribution of the vibrato extent and rate in relation to *jo*.

Table 1 presents the means, standard deviations, and results of a skewness analysis of the acoustic features of vibratos in Kim Juk-pa’s *sanjo* performance in relation to the *jo* type and the melodic line. The number of instantaneous values for each vibrato, in terms of its rate and extent, varied as a function of the number of identified crests and troughs. The mean values for vibrato extent in relation to jo type were organized as *g-jo*, *u-jo*, and *p-jo*, while those for vibrato rate were organized as *p-jo*, *g-jo*, and *u-jo*. The acoustic parameters of the vibrato segments related to the melodic lines were found to be consistent in ascending, descending, and static order. Figure 1 shows the distribution of the acoustic parameters of the observed vibratos.

**Table 1:** Descriptive analysis of vibrato extent and vibrato rate across *jo* type and melodic line.

Variable	Category	Vibrato extent (st)				Vibrato rate (Hz)			
		*N*	*M*	*SD*	*Sk*	*N*	*M*	*SD*	*Sk*
*Jo*	*U-jo*	221	1.805	0.607	−0.171	221	5.047	0.755	2.174
	*P-jo*	46	1.190	0.332	−0.394	46	5.281	0.640	−0.180
	*G-jo*	516	2.686	0.847	−0.207	516	5.249	0.700	2.605
Melodic line	Ascending	405	2.524	0.910	−0.114	405	5.297	0.690	2.040
	Descending	101	2.301	0.923	0.458	101	5.089	0.724	0.464
	Static	277	2.111	0.837	0.237	277	5.081	0.735	3.413

The melodic line, defined as the sequential progression of pitch events, is categorized into three distinct contour patterns: ascending, descending, and static. The *jo* types include *u-jo* (majestic), *p-jo* (peaceful or joyful), and *g-jo* (melancholic or sad).

Significant differences owing to the interaction between *jo* and melodic line were found for vibrato extent (*p* < 0.001) and vibrato rate (*p* < 0.001). Partial eta-squared indicated the effect of *jo* on the vibrato extent (η2p = 0.289) and rate (η2p = 0.017), as shown in Table 2. Furthermore, significant differences between vibrato extent and vibrato rate were found across the three different features of the melodic lines (*p* < 0.001).

**Table 2:** Vibrato extent and vibrato rate across *jo* and melodic line.

Variable	Acoustic parameter	Source	*df*	*SS*	*MS*	*F*	*p*	*η^2^_p_*
*Jo*	Vibrato extent (st)	Between groups	2	185.616	92.808	158.884	0.001	0.289
		Within groups	780	455.617	0.584			
		Total	782	641.233				
	Vibrato rate (Hz)	Between groups	2	6.739	3.369	6.634	0.003	0.017
		Within groups	780	396.144	0.508			
		Total	782	402.883				
Melodic line	Vibrato extent (st)	Between groups	2	28.315	14.158	18.017	0.001	0.044
		Within groups	780	612.918	0.786			
		Total	782	641.233				
	Vibrato rate (Hz)	Between groups	2	8.939	4.470	8.850	0.001	0.022
		Within groups	780	393.944	0.505			
		Total	782	402.883				

A Games–Howell test revealed significant differences in the acoustic parameter values of vibrato segments across the three types of *jo* and melodic lines. Multiple comparisons yielded significant findings related to *jo* (*p* < 0.001) for both vibrato extent and vibrato rate, particularly for *u-jo* and *g-jo* (*p* < 0.001). In terms of melodic lines, notable disparities in the vibrato extent and vibrato rate were observed between ascending and static structures (*p* < 0.001). Significant differences in vibrato rate also emerged between ascending and descending structures (*p* < 0.001).

The descriptive statistical analysis of the transformed values for vibrato extent and vibrato rate in relation to *jo* and melodic line is detailed in Table 3. Because of the inadequate sample size of the vibrato segments associated with *p-jo*—as a structural feature in Kim Juk-pa’s *sanjo* performance—these vibrato segments were excluded from the analysis. Additional analyses revealed no effect from the interaction between *jo* type and melodic line—that is, the combined effect of *jo* and melodic line on the acoustic parameters vibrato extent and vibrato rate (Tables 4, 5). However, both the *jo* type and the melodic line were found to have a significant main effect on vibrato rate (*p* < 0.001) and vibrato extent (*p* < 0.05).

**Table 3:** Descriptive analysis of vibrato rate and vibrato extent.

Acoustic parameter	*U-jo*						*G-jo*					
	Ascending		Descending		Static		Ascending		Descending		Static	
	*M*	*SD*	*M*	*SD*	*M*	*SD*	*M*	*SD*	*M*	*SD*	*M*	*SD*
$\sqrt{\mathrm{ve}}$	1.359	0.246	1.295	0.228	1.272	0.259	1.659	0.292	1.681	0.249	1.530	0.260
$\sqrt{vr^{3}}$	11.712	2.994	11.641	2.973	10.709	1.724	12.475	2.325	11.490	1.892	11.720	3.027

Ve indicates vibrato extent and vr denotes vibrato rate.

**Table 4:** Two-way factorial ANOVA results for vibrato extent transformed to $\sqrt{\mathrm{ve}.}$

Variable	*SS*	*df*	*MS*	*F*	*p*	*η^2^_p_*
*Jo*	11.966	1	11.966	166.930	0.001	0.186
Melodic line	1.362	2	0.681	9.498	0.001	0.025
*Jo ×* melodic line	0.261	2	0.130	1.818	0.163	0.005
Residual	52.402	731	0.072			

**Table 5:** Two-way factorial ANOVA results for vibrato rate transformed to $\sqrt{vr^{3}}$ .

Source	*SS*	*df*	*MS*	*F*	*p*	*η^2^_p_*
*Jo*	35.367	1	35.367	5.184	0.023	0.007
Melodic line	92.228	2	46.114	6.760	0.001	0.018
*Jo ×* melodic line	22.915	2	11.457	1.679	0.187	0.005
Residual	4986.824	731	6.822			

**Figure 2:**
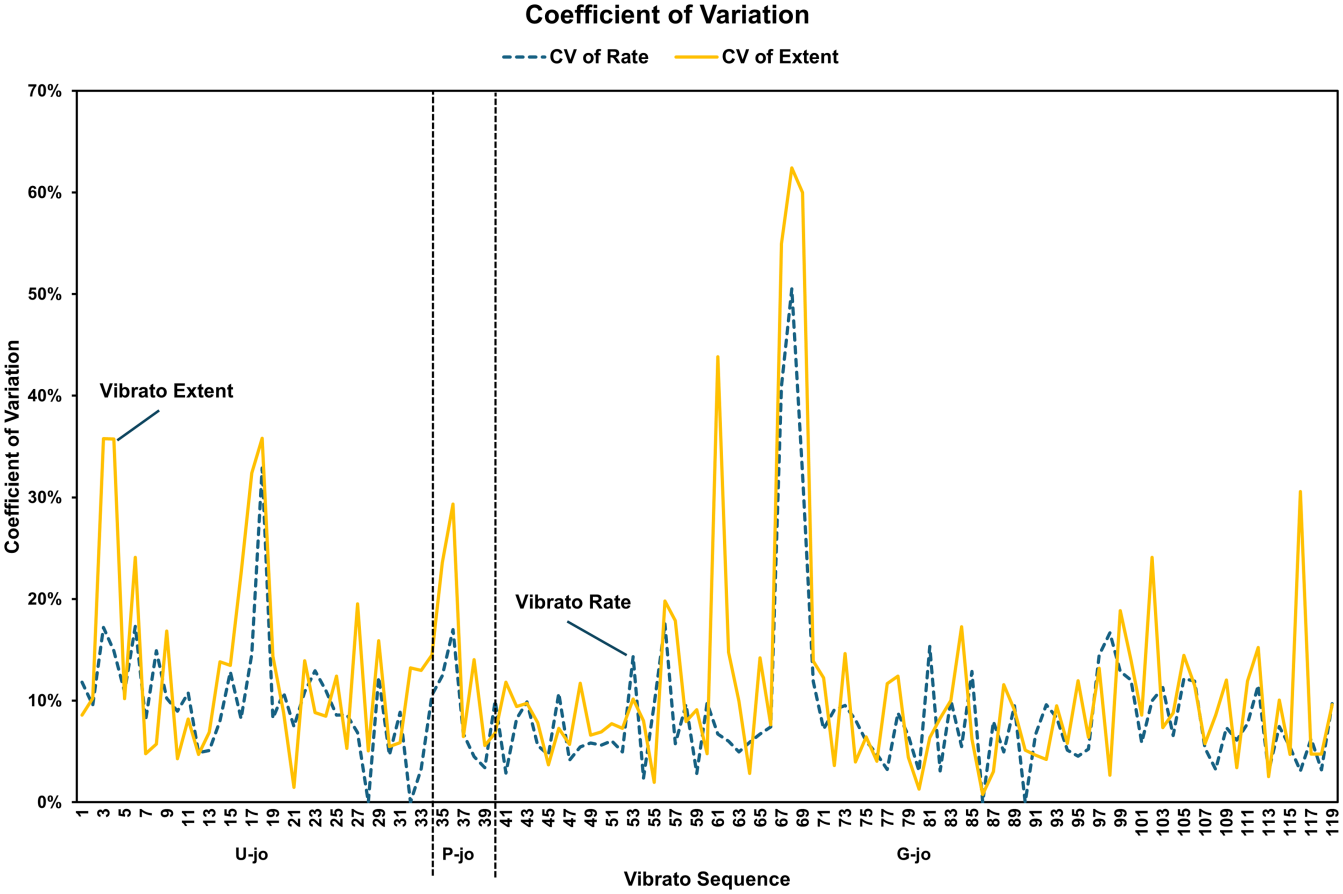
Visualization of the coefficient of variance (CV) distribution for vibrato rate and vibrato extent as a function of *jo*.

The coefficient of variance (CV) for both the vibrato rate and vibrato extent reflects the normalized values of the vibrato parameters. The mean values of vibrato extent were consistently higher than those of vibrato rate, regardless of *jo* type or melodic line (Table 6), and no significant differences were found between the CV for vibrato extent and vibrato rate across the different *jo* types and melodic features (Table 7). Furthermore, there was a strong association between the CV values for vibrato rate and vibrato extent, varying systematically according to *p-jo*, *g-jo*, and *u-jo* (*p* < 0.001), as well as descending, ascending, and static melodic lines (*p* < 0.001). Inconsistent variance at specific points in *u-jo* and *g-jo* are highlighted in Figure 2, with considerable differences in magnitude observed across *jo* types. However, the similar CV values for vibrato extent and vibrato rate suggest a relatively consistent relationship between the CV values of the vibrato parameters.

**Table 6:** Descriptive analysis of the coefficient of variance for vibrato extent and vibrato rate.

Variable	Category	Coefficient of variance for vibrato extent				Coefficient of variance for vibrato rate			
		*N*	*M*	*SD*	*Sk*	*N*	*M*	*SD*	*Sk*
*Jo*	*U-jo*	33	0.135	0.097	1.279	33	0.102	0.058	1.649
	*P-jo*	6	0.156	0.094	0.493	6	0.091	0.052	0.504
	*G-jo*	80	0.114	0.115	3.122	80	0.087	0.075	3.573
Melodic line	Ascending	59	0.109	0.090	3.323	59	0.084	0.056	2.822
	Descending	38	0.121	0.100	2.599	38	0.097	0.067	2.695
	Static	22	0.155	0.158	1.687	22	0.101	0.102	3.124

**Table 7:** Coefficient of variance for vibrato extent and vibrato rate across jo and melodic line.

Variable	CV	Source	*df*	*SS*	*MS*	*F*	*p*	*η^2^_p_*
*Jo*	CV for vibrato extent	Between groups	2	0.018	0.009	0.760	0.470	0.013
		Within groups	116	1.384	0.012			
		Total	118	1.402				
	CV for the vibrato rate	Between groups	2	0.005	0.002	0.498	0.609	0.009
		Within groups	116	0.570	0.005			
		Total	118	0.575				
Melodic line	CV for the vibrato extent	Between groups	2	0.034	0.017	1.438	0.242	0.024
		Within groups	116	1.368	0.012			
		Total	118	1.402				
	CV for the vibrato rate	Between groups	2	0.006	0.003	0.652	0.523	0.011
		Within groups	116	0.569	0.005			
		Total	118	0.575				

### 5.2 Phase 2: performers’ reflections and qualitative interpretation

#### 5.2.1 Participants

Considering the focus of this study on a highly specialized musical tradition (i.e., *sanjo*), purposive sampling was employed to recruit participants with deep domain-specific expertise. Five professional *sanjo* performers (P1–P5) participated in the study. All participants specialized in the 12-string *gayageum sanjo* tradition and possessed substantial professional experience, ranging from 12 to 18 years of active performance in *gayageum*. All participants had commonly studied Kim Juk-pa *sanjo*, one of the most historically influential and pedagogically established *sanjo* lineages, and each performer had received training directly from disciples of Kim Juk-pa, ensuring authentic transmission of stylistic knowledge. Their years of experience performing the Kim Juk-pa *sanjo* repertoire ranged from 2 to 18 years, reflecting both long-term lineage bearers and relatively newer practitioners who had incorporated the style into their developing artistic identity. Their backgrounds contribute to gaining broader insights of using MIR based *sanjo* learning such as nuanced reflections on vibrato use, stylistic interpretation, and expressive decision-making. Their professional roles reflected a diverse range of career pathways within the traditional music field, including institutional instruction, orchestra membership, freelance teaching, and graduate-level performance training. To ensure confidentiality, all identifying details were anonymized and participants are referred to using coded labels (P1–P5) in accordance with IRB guidelines.

#### 5.2.2 Procedure

To examine the participants’ perspectives on the quantitative findings—particularly their relevance to *sanjo* vibrato practice—a group interview was conducted with purposefully selected professional *sanjo* performers. Purposive sampling ensured the inclusion of participants with extensive knowledge, advanced technical skills, and substantial experience in *sanjo* performance (Etikan et al., 2016). From a methodological perspective, the group interview was employed to facilitate the efficient emergence of shared reference points, areas of convergence, and points of divergence among participants, thereby enabling the identification of salient themes across the group without foregrounding individual case-based comparisons. Also, in focus group–based qualitative research, a group size of approximately four to six participants is widely regarded as optimal for facilitating balanced participation, sustained engagement, and the emergence of diverse perspectives without fragmenting the discussion.

Prior to the Phase 2 group interview, a preparatory meeting was conducted to support performers’ informed engagement with the MIR-based performance analyses and to enhance the trustworthiness of the study. During this meeting, performers were provided with a structured document that sequentially introduced the analytical materials and their interpretive relevance, including quantitative tables, graphical visualizations, and correlational summaries, together with explanatory descriptions of the results as established in Phase 1.

The preparatory materials included, tabulated summaries of vibrato depth and vibrato rate across modal contexts (*u-jo, p-jo, g-jo*) and melodic motion types (ascending, descending, and stable phrases). These tables were accompanied by verbal explanations clarifying what each numerical index represented (e.g., mean vibrato depth as an indicator of pitch excursion, vibrato rate as temporal fluctuation). In these figures, performers were guided through the meaning of axes, line trajectories, and points at which vibrato depth and rate diverged in direction. For example, particular segments were highlighted in which vibrato depth increased while vibrato rate decreased, prompting discussion about moments where expressive emphasis might be achieved through depth rather than speed. Also, visual graphs and the correlational relationship between the variability of vibrato depth and vibrato rate were introduced to illustrate how vibrato depth and rate unfolded sequentially across phrases.

From a methodological perspective, the group interview allowed common points of reference, convergence, and divergence to surface efficiently, thereby supporting the identification of salient themes across participants without prioritizing individual case comparisons. In the group meeting, performers were encouraged to relate these summaries to their prior experiential knowledge of modal affect and melodic shaping rather than to treat the values as prescriptive norms. The question examples provided were as follows:

“Do the numerical summaries of vibrato depth and vibrato rate across modal categories and melodic motion types offer learning-relevant information beyond what is typically discussed in conventional lessons? Please describe how such information may contribute to the refinement of your expressive practice.”

“When looking at the graph, how do you interpret the sections where vibrato depth and vibrato rate move in opposite directions where depth increases while rate decreases? Do these visual patterns help you reflect on expressive emphasis?”

“The analysis shows associations between the variability of vibrato depth and the variability of vibrato rate. Does this relationship align with your experiential understanding of vibrato practice in Kim Juk-pa’s *sanjo*? If so, how do you interpret this relationship in your own performance?”

Semi-structured prompts encouraged participants to articulate detailed and nuanced accounts of how they interpreted and evaluated the Phase 1 findings. The interview transcripts were systematically coded, and emerging themes were identified through an iterative process of review and discussion between the researchers following established guidelines for ensuring the rigor and trustworthiness of thematic analysis (Nowell et al., 2017).

## 6 Qualitative findings

The thematic analysis resulted in five interconnected themes that illuminate how professional *sanjo* performers interpreted and engaged with the quantitative vibrato findings. The themes reflect core dynamics of apprenticeship-based musical learning, including lineage transmission, reflective re-engagement with stylistic archetypes, and the negotiation of personal artistic identity.

### 6.1 Theme 1: quantified vibrato as an explicit confirmation of tacit, embodied lineage learning

Participants emphasized that *sanjo* vibrato is traditionally learned through embodied, apprenticeship-based processes—that is, through imitation, motor practice, and close observation of teachers rather than through written notation. Against this backdrop, the quantitative vibrato profiles provided a rare form of explicit, externalized evidence that made their tacit, bodily knowledge visible and verifiable. The numerical patterns allowed performers to confirm that the techniques they had internalized through long-term practice aligned with the vibrato characteristics exemplified in Kim Juk-pa’s performance. As P5 noted, “Seeing it in direct numerical terms gave me more confidence in what I played,” and similarly P4 remarked, “Seeing it this way gave me confidence that what I learned was correct.”

This alignment was articulated with particular clarity by P1, who reflected:

“I share that perception. In my training, even in the case of stable melodic tones—such as sustained main tones or less ornamented pitches—vibrato was expected to be applied continuously. From that perspective, this practice appears to correspond closely with the patterns observed in the numerical data.”

Phase 1 did not interact with or modify the performers’ embodied learning itself. Rather, it provided an explicit, externalized representation of the vibrato features they had already internalized through bodily practice. By making these tacit techniques visible in measurable form, the analysis enabled participants to recognize correspondences between their embodied skills and the master’s vibrato characteristics, thereby reinforcing their confidence in the accuracy and authenticity of their lineage-based training.

### 6.2 Theme 2: quantitative analysis as a catalyst for heightened attentional engagement with the stylistic archetype

Beyond confirming what they had previously learned, the vibrato profile served as a reflective catalyst that prompted performers to re-engage with foundational aspects of the stylistic archetype. Several participants described the analysis as enabling more attentional focus on expressive features that had either faded from conscious awareness or had not been explicitly examined over time. For instance, P2 described:

“I had somewhat forgotten this. In particular, regarding melodic characteristics, I had assumed that the average vibrato speed would not differ substantially between descending melodies and stable melodies. However, when I examined the detailed numerical data, I realized that there were in fact clear differences, which I was able to compare directly. Comparing what I heard by ear with the numerical analysis allowed me to approach these subtle distinctions with greater precision.”

In the similar context, P4 explained:

“In areas where I previously struggled, such as vibrato, I think I can now gain more support by listening to the recordings while also referring to the charts and thinking, ‘This is how it should be done.’ Before, I relied solely on listening, but being able to verify these features visually through numerical data will help me practice with greater clarity.”

Participants reported a growing recognition that their previously more implicit modes of learning could accommodate elements of more conscious engagement. Although they did not articulate concrete or fully developed strategies for doing so, the experience of engaging with performance data translated into visualized form prompted serious reflection on how they might think more deliberately and take greater initiative in their own learning processes. Working with quantified representations of their performance encouraged performers to consider more actively on refinement of their practice.

### 6.3 Theme 3: cognitive unfamiliarity and the need for pedagogical scaffolding

Although participants recognized the analytical value of the vibrato data, many reported an initial sense of discomfort or cognitive friction when engaging with the numerical representations. In contrast to the oral–aural and embodied modes of transmission that characterize *sanjo* pedagogy, numerically summarized visualizations required interpretive strategies that fall outside performers’ customary learning ecology. This tension was articulated by P5, who described the chart as feeling “somewhat standardized,” noting that “if the gap between how the data are generated and the form in which performers can use them were reduced, it would become a very valuable resource; however, at this stage, it still seems somewhat difficult to understand.” Similarly, P2 remarked that “it doesn’t immediately stand out to me,” “It would be even better if this research could expand to include different versions from various years, allowing for comparisons across those as well,” underscoring the interpretive adjustments required to make sense of the figures. Across participants, there was broad agreement that closer integration of the numerical data with musical notation would provide essential scaffolding for interpretation, as P1 observed: “If this chart were presented alongside the score, it would be easier to understand.” And P4 pointed out: I thought that if this chart had been presented alongside the sheet music, it might have been more helpful in understanding the chart, especially if certain parts were marked with the music. These reflections highlight a representational gap between analytic visualization of music performance data and its modes of musically or pedagogically grounded sense-making.

### 6.4 Theme 4: quantitative data as a catalyst for micro-level expressive articulation

Participants described concrete ways and individualized focus in which the vibrato analysis informed micro-level refinement of their expressive technique. The numerical distinctions between vibrato depth and rate offered a level of precision that performers do not typically access through listening alone. With this information, they could identify specific passages where the two parameters diverged in subtle but musically significant ways.

P2 noted:

“If I were to refine my expressiveness using this chart, I would focus on the sections where the depth and speed of the vibrato go in opposite directions. By listening to the vibrato in sections 46, 67, and 69 and comparing it with this chart, I would attend to how the depth and speed of the vibrato are expressed in contrast, and I would refine my expressiveness with that in mind. Unexpectedly the rapid vibrato in melodic contexts they had assumed to be stable broadened my awareness of expressive nuance.”

P1 revealed:

“I would focus on sections where the depth and speed move in opposite directions and refine my expressiveness with that in mind. I think it would be helpful to identify and check those aspects.”

These insights demonstrate the role of quantitative analysis as a micro-diagnostic resource within expert performance learning. The analysis made tacit, fine-grained aspects of vibrato perceptually explicit and supported more deliberate and precise adjustment by performers. The approach aligns with mastery-oriented learning processes, where improvement occurs through repeated cycles of focused attention, perceptual clarification, and intentional technical refinement. The vibrato profiles offered more than a description of the master’s technique; they provided performers with a structured tool for examining and elevating the subtleties of their own expressive practice.

### 6.5 Theme 5: negotiating fidelity to lineage, teacher influence, and performers’ agency

This category was developed to foreground performers’ recognition of an ongoing tension between fidelity to artistic lineage and the interpretive influence of their current artist-teachers, as well as their emerging awareness of themselves as active agents capable of mediating this tension. While these issues were not entirely unfamiliar to the participants, engagement with the MIR-based vibrato analysis rendered their awareness of multiple sources of authority more explicit and consciously articulated.

The vibrato analysis sharpened participants’ thoughts of the complex ongoing negotiation in terms of the interpretive imprint of their present artist-teachers. Participants described considerable variation in how different teachers emphasized—or at times de-emphasized—the “original form,” resulting in inconsistencies in how the lineage was transmitted across pedagogical contexts. P1 and P5 reflected on this variability:

“I’ve studied the Kim Juk-pa style with many teachers, and because I’ve studied it for so long, I’ve learned from so many teachers. However, many of those teachers, despite their extensive studies, didn’t focus much on the original form, which often led to lessons that didn’t address that aspect. I think if the focus on the original form had been established earlier, I might have been able to study more comfortably. Also, as we continue studying, we encounter many great performers, and sometimes, I feel that the original form tends to be overlooked …”

“I have mainly focused on the Kim Juk-pa style. I learned from various teachers since middle school, and what I noticed is that while they all had the goal of following the original form, they sometimes strayed from it in order to express their own musicality. However, in the end, they all seemed to return to seeking the original form.”

These reflections indicate that lineage transmission was experienced not as a stable or uniform process, but as one shaped by successive layers of pedagogical interpretation. P4’s account further articulated this emerging awareness by explicitly linking it to engagement with MIR-based performance data. P4 emphasized how analytical access enabled a reflective return to the stylistic archetype and supported more deliberate interpretive positioning within their current music performance learning:

“Since *sanjo* is music that was passed down orally rather than written, it has changed a lot depending on the performer’s skill, and in some ways, it may have even been altered. In this situation, I think it was a great opportunity to return to the roots of the music, understand it, and analyze it again. In classical music, for example, composers clearly pursue and intentionally express certain aspects, and those intentions are conveyed to future generations. But in the case of Kim Juk-pa, the specific intentions or expressive elements that he wanted to emphasize may not have been clearly transmitted. Through this type of music performance data, I think we can more clearly understand what the virtuoso intended or wanted to express [under the influence of our current teachers].”

## 7 Discussion

This study examined how MIR-based vibrato analysis from a historically significant recording by Kim Juk-pa becomes meaningful within the intertwined cognitive, cultural, and pedagogical ecologies of lineage-based apprenticeship. By integrating computational profiling (Phase 1) with performers’ reflective interpretations (Phase 2), the findings reveal that acoustic measurements do not function merely as objective descriptors of expressive behavior but acquire relevance and interpretive force when situated within the cultural logics, embodied practices, and lineage-specific epistemologies through which a traditional music form is transmitted. The interplay between quantitative representations and enculturated musicianship demonstrates that performance analysis, when embedded in culturally coherent learning systems, can illuminate expressive structures that are otherwise tacit, intuitive, or only partially verbalized.

The vibrato profiling showed structured variability across *jo* types, offering insight into the expressive architecture of the *sanjo* lineage examined here. This structured pattern—particularly the association with vibrato extent and vibrato rate at the level of variance rather than mean—supports theoretical models conceptualizing expressive individuality as an emergent property of coordinated acoustic cues rather than isolated parameters (Leech-Wilkinson, 2009; Timmers and Desain, 2000). These findings also resonate with ethnomusicological scholarship emphasizing that expressive nuance in traditional musics is culturally encoded, historically situated, and learned through lineage-specific aesthetic vocabularies (Blacking, 1973; Howard, 2012). In this sense, the acoustic features traced through MIR do not serve as universal affective markers but appear embedded in a culturally grounded expressive semantics shaped by shared listening practices, genre-specific idioms, and long-standing performer–listener conventions.

The visualized vibrato profiles functioned as an interpretive augmentation of close listening, rendering aspects of the virtuoso’s expressive logic perceptually salient without claiming epistemic priority over culturally grounded modes of musical understanding. In this sense, the present approach accords with performance studies scholarship that conceives analytical and visualization-based representations not as substitutes for listening, but as reflexive tools capable of orienting attention toward patterned expressive structures that often remain implicit within unaided auditory experience, particularly at the micro-temporal level (Cook, 2013; Leech-Wilkinson, 2009). Such representations make it possible to examine micro-level nuances in phrasing, timing, and timbre that would remain tacit within orally transmitted traditions, thereby complementing embodied and culturally grounded modes of learning. By externalizing micro-expressive nuances that would otherwise be tacit or ephemeral, the profiles allowed performers to integrate culturally inherited understandings with analytically derived insights, enriching their apprehension of lineage-specific expressivity. The decoding of vibrato shaping in this study emerged through a dialogic process in which cultural knowledge, embodied experience, and computational representation converged to support a deeper understanding of Kim Juk-pa’s expressive vocabulary defined as individuality.

Traditional *sanjo* training is embedded in a master–apprentice system in which expressive knowledge is transmitted through embodied imitation, close interpersonal interaction, and repeated exposure to lineage-specific exemplars. Although the specific musical content and cultural foundations differ across traditions, the underlying pedagogical principles resonate with broader ethnographic and performance studies demonstrating that orally transmitted musics rely heavily on tacit, body-based learning and situated mentorship (Berliner, 1994; Green, 2002). Within this framework, performers described the profiles as clarifying aspects of expressive technique that had previously been sensed. This transformation of tacit impressions into explicit acoustic information clarifies aspects of performance that are often difficult to articulate or to monitor reliably through listening alone. In the present study, the MIR output functioned as a cognitive scaffold that made implicit lineage knowledge more accessible for reflection, while expressive control continued to develop through embodied practice. The findings suggest that annotated music performance profiling can operate as interpretive mediators within apprenticeship systems and enhance performers’ reflective engagement in their learning process.

A further pattern emerging from performers’ reflections concerns the role of analysis in stylistic recentering. Recognizing the diversity of music teaching and learning as embedded within distinct musical cultures (Rice, 2003), prior research on music tuition in higher education has emphasized performers’ reflective engagement with interpretive divergence, in which stylistic models are mediated, refracted, and rearticulated through pedagogical interaction rather than transmitted intact (Kim, 2016; Zhukov, 2007). In contrast to such instructor-mediated processes, performers in this study described the vibrato profiles as facilitating a more direct analytical encounter with the virtuoso’s expressive practice itself. Rather than relying on contemporary pedagogical filters, the profiles enabled performers to recalibrate their expressive aims toward the sonic archetype instantiated in Kim Juk-pa’s archival performance. This process in a sense resembles mastery-oriented learning, in which musicians periodically realign interpretive strategies through deliberate practice and targeted refinement against authoritative standards (Ericsson et al., 1993). Although participants remained situated within present-day apprenticeship contexts, the use of MIR-based vibrato profiling functioned as a technical and perceptual resource that supplemented and supported pedagogical mediation—including teachers’ stylistic exemplification, and interpretive emphasis—by providing performers with direct access to data-driven representations of lineage-defining expressive features.

The profiles also supported micro-expressive refinement by enabling performers to identify subtle divergences in vibrato depth, rate, and their interaction—parameters that are often registered tacitly but remain difficult to monitor with precision through auditory attention alone, particularly during active performance. This limitation reflects a broader constraint in performers’ perceptual access to fine-grained temporal and parametric variation, whereby certain micro-level deviations fall below the threshold of reliable conscious detection. From this perspective, the use of vibrato profiling compensates for known perceptual boundaries in expressive monitoring, a point consistent with evidence that sensitivity to minute temporal variations in performance is inherently constrained (Repp, 1999). Other studies in expressive-performance research show that visualized analytic representations can make micro-level expressive variation more perceptually and analytically tractable that support reflective comparison and fine-tuning rather than replacing musical judgment or interpretive agency (Langner and Goebl, 2003; Widmer and Goebl, 2004). While a few participants expressed some ambivalence about the risk of over-precision leading to stylistic rigidity, the majority recognized that, when used cautiously and interpretively, analytic annotation can enhance expressive intentionality and facilitate nuanced control—suggesting that sound knowledge grounded annotation may enrich artistic agency when embedded within pedagogical environments that remain flexible and interpretively open.

A recurring concern among performers involved negotiating the interplay between fidelity to an ancestral style, the mediating influence of their immediate teachers, and the cultivation of a personal artistic identity. The vibrato profiles heightened performers’ awareness of this tension by rendering lineage-defining expressive features more explicit, thereby clarifying the interpretive boundaries within which individual artistry could be developed. This dynamic resonates with prior observations that analytic or score-based frameworks can simultaneously support interpretive insight and delimit expressive latitude. In the present context, however, performers tended to appropriate the profiles not as prescriptive constraints but as orienting landmarks within a broader expressive terrain, using them to navigate the balance between obligations to their current knowledge and the pursuit of individual creativity interplaying with the virtuoso’s. As Burwell (2012) notes, apprenticeship-based learning ultimately aims to foster performers’ musical confidence and interpretive independence. From this perspective, the integrated form of musical text as suggested by the performers—balancing transcription-based analytical representation with conventional notation—may open an additional dimension of musical agency by providing performers with a structured yet negotiable reference point observed in the archetype form for interpretive decision-making.

Taken as a whole, the findings suggest an integrative account of how computational analysis interfaces with lineage-based musicianship. MIR-derived profiles externalize tacit expressive structures; performers interpret these structures through musically grounded schemas and embodied learning; and reflective engagement with profiles reshapes expressive development in ways that strengthen stylistic centering, heighten perceptual discrimination, and refine personal artistry. This reciprocal dynamic resonates with cognitive models proposing that expert performance emerges through the integration of explicit conceptual knowledge, perceptual learning, episodic memory, and motor expertise. In this study, the MIR-based profiles strengthened the explicit and perceptual dimensions of this system, while performers’ lineage-based training contributed episodic memory and motor expertise; together, these components expanded the possibilities for achieving more refined expressive control. It also supports interdisciplinary efforts to situate computational analysis within ethnographic, pedagogical, and cultural contexts, illustrating how quantitative tools can enrich rather than displace the humanistic dimensions of performance learning.

Taken as a whole, the findings point to an integrative account of how computational analysis interfaces with lineage-based musicianship. MIR-derived vibrato profiles externalize tacit expressive structures that are typically absorbed through prolonged apprenticeship, making them available for reflective scrutiny. Performers interpret these structures through musically grounded schemas shaped by embodied learning, and sustained engagement with the profiles in ways that reinforce stylistic centering, heighten perceptual discrimination, and refine artistic insights. Moreover, this process affords performers a degree of interpretive flexibility by loosening their exclusive reliance on the temporal and pedagogical constraints of their current apprenticeship although there were limitations associated with performers’ unfamiliarity or discomfort with numerical and parameterized representations of vibrato. For example, for some participants, engaging with quantified profiles of vibrato variability required an additional layer of interpretive translation before the data could be meaningfully integrated into musical decision-making. This implies that the value of vibrato profiling depends on performers’ ability to relate analytic outputs to embodied sensation, auditory imagery, and stylistic knowledge. Such reciprocal dynamic resonates with cognitive accounts of expert performance in which expressive mastery emerges through the integration of explicit conceptual knowledge, perceptual learning, and motor expertise.

## 8 Conclusion

In the present study, MIR-based analytical profiles primarily served to validate and make explicit key perceptual dimensions of performers’ lineage-based training. When engaged reflectively, these representations expanded performers’ capacity for more finely differentiated and self-directed expressive control. More broadly, the findings support interdisciplinary efforts to situate computational analysis within ethnographic and pedagogical frameworks, demonstrating that quantitative tools can extend—rather than displace—the humanistic dimensions of performance learning when they are appropriately mediated. Within Korean traditional music and other lineage-based systems, master recordings continue to function as central cultural resources through which expressive identity is transmitted. The present study demonstrates that MIR-based analysis can deepen performers’ engagement with these authoritative sonic models, providing a reflective foundation for informed and coherent musical creativity. At the same time, revisiting stylistic archetypes such as *sanjo* may generate tensions with performers’ current pedagogical inheritances, as reflected in participants’ accounts. Such tensions are not anomalous but inherent to apprenticeship-based traditions, in which multiple layers of lineage authority coexist and, at times, compete. Importantly, this study suggests that re-engagement with foundational aesthetic models is generative rather than constraining. By confronting earlier stylistic ideals through analytical reflection, performers may encounter productive friction that stimulates critical self-positioning and supports the further articulation of artistic individuality. While the present study did not pursue a fine-grained analysis of interactional processes among performers, it focused instead on the substance of performers’ expressed perceptions of analytical representations. Future research may extend this work by examining how such perceptions are collectively negotiated and transformed through interaction over a period of time.

## Data Availability

The raw data supporting the conclusions of this article will be made available by the authors, without undue reservation.

## Author’s note

A version of Kim Juk-pa’s *sanjo* performance is publicly available on YouTube at the following link: https://www.youtube.com/watch?v=aqhA3nLSfeE&list=RDaqhA3nLSfeE&start_radio=1.
